# Chloroform Treatment for Hemicrania and Neuralgia

**Published:** 1852-10

**Authors:** 


*Chloroform Ointment for Hemicrania and Neuralgia.*

M. Cazenave, of Bordeaux, recommends the above ointment, which is prepared as follows:—Pure chloroform, three drachms; cyanide of potassium, two drachms and a half; axunge, two ounces; add a sufficient quantity of white wax to make an ointment of the usual consistence.—*Ibid.*

